# Results From a Phase 1 Study Evaluating the Safety, Tolerability, Pharmacokinetics, Pharmacodynamics, and Efficacy of ANX005, a C1q Inhibitor, in Patients With Guillain–Barré Syndrome

**DOI:** 10.1111/jns.70009

**Published:** 2025-02-25

**Authors:** Quazi Deen Mohammad, Zhahirul Islam, Nowshin Papri, Shoma Hayat, Israt Jahan, Khan Abul Kalam Azad, Dean R. Artis, Benjamin Hoehn, Eric Humphriss, Ping Lin, Ted Yednock, Henk‐André Kroon

**Affiliations:** ^1^ National Institute of Neurosciences & Hospital Dhaka Bangladesh; ^2^ Laboratory of Gut–Brain Axis, icddr,b Dhaka Bangladesh; ^3^ Department of Neurology Erasmus MC, University Medical Center Rotterdam the Netherlands; ^4^ Dhaka Medical College and Hospital Dhaka Bangladesh; ^5^ Annexon Bioscience Brisbane California USA

**Keywords:** ANX005, C1q, complement, Guillain–Barré syndrome, Phase 1

## Abstract

**Background and Aims:**

Guillain–Barré syndrome (GBS) is an acute autoimmune peripheral neuropathy driven by autoantibodies and classical complement pathway activation. Despite treatments with intravenous immunoglobulin or plasma exchange, GBS remains characterized by variability in recovery and high incidence of residual disabilities. This randomized, double‐blind, placebo‐controlled Phase 1 trial evaluated the safety, tolerability, and pharmacokinetics of ANX005, a novel therapeutic targeting the classical complement cascade.

**Methods:**

Patients with recent‐onset GBS, who had no access to intravenous immunoglobulin or plasma exchange, received escalating doses of ANX005 or placebo as a single IV infusion. Primary objectives included assessments of safety. Secondary objectives included determination of pharmacokinetic and pharmacodynamic profiles and clinical outcomes through Week 8. Exploratory objectives included an evaluation of serum and cerebrospinal fluid (CSF) complement and tissue damage biomarkers.

**Results:**

Fifty patients were randomized to receive either ANX005 (*n* = 38) or placebo (*n* = 12). ANX005 was well‐tolerated across all doses with no dose‐limiting toxicities, suggesting an acceptable safety profile. Pharmacodynamic data showed effective C1q inhibition and a reduction in neurofilament light chain levels, suggesting nerve damage mitigation. Exploratory endpoints evaluating clinical outcomes included improvements in Medical Research Council sum scores, GBS‐Disability Score, and Overall Neuropathy Limitations Scale with ANX005 compared to placebo, particularly in patients receiving doses that inhibited serum C1q for ≥ 1 week and provided C1q blockade in the CSF.

**Interpretation:**

These results support ANX005 as a targeted therapy for GBS that modulates the classical complement pathway. Further investigation in a larger Phase 3 trial is warranted to confirm these results and assess the long‐term benefits of complement inhibition in patients with GBS.

## 1 Introduction

Guillain–Barré syndrome (GBS) is a rare, acute, and severe life‐threatening paralytic inflammatory autoimmune neuropathy of the peripheral nervous system (PNS) [1, 2, 3]. The syndrome is characterized by rapidly progressive, monophasic antibody‐mediated injury of peripheral nerves typically following an antecedent infection [1]. Over a period of 2–4 weeks, nerve damage progresses until titers of the cross‐reactive, complement‐activating antibodies have diminished [2].

Muscle weakness is the primary manifestation of GBS, and the severity of muscle weakness at diagnosis and at nadir, as measured by Medical Research Council (MRC) sum score, is highly variable [1, 4]. Despite the observed variability, muscle strength is highly prognostic of GBS outcomes and long‐term disability when assessed both at presentation and after 1 week [5]. Even when treated with intravenous immunoglobulin (IVIg) or plasma exchange (PE), approximately 25% of patients demonstrate worsening muscle weakness after treatment, and 10% of patients deteriorate after an initial improvement [1]. Nearly 25% of patients develop respiratory insufficiency requiring artificial ventilation and are at particular risk of mortality, which ranges from 3% to 17% in the first year after diagnosis [2, 6, 7, 8, 9].

IVIg and PE were introduced over 30 years ago based on their ability to hasten recovery during the acute and subacute stages of GBS [8, 10]. No alternative PE or IVIg regimens have been shown to significantly improve the course of GBS [11]. Therefore, the standard of care continues to be PE and IVIg [12], neither of which have a targeted mechanism of action.

In GBS, antibodies created by an adaptive immune response against particular pathogens cross‐react with self‐components of peripheral nerves, recruiting C1q to the nerve surface for activation of the classical complement pathway [13]. IgM and IgG anti‐ganglioside antibodies and increased complement activity can be observed in the cerebrospinal fluid (CSF), consistent with breakdown of the blood–nerve barrier in the early stages of GBS [14]. C1q binding triggers activation of the classical complement pathway along with surface deposition of C4b, C3b, and C5b‐9 as well as solution‐phase release of the anaphylatoxins C3a and C5a [15]. The anaphylatoxins recruit and activate immune cells, particularly, macrophages within peripheral nerve tissue, which attack nerve surfaces that have been coated with complement activation products [16]. Further amplification of the classical complement pathway leads to the formation of the terminal complement complex (C5b‐9), which results in direct membrane damage of myelin (acute inflammatory demyelinating polyneuropathy [AIDP]) and axons (acute motor axonal neuropathy [AMAN]) within the peripheral nerve [15, 17, 18]. Complement‐mediated neuroinflammation and peripheral nerve damage lead to muscle weakness or paralysis of arms and legs [8, 13, 15]. Blocking the ongoing binding of C1q and activation of the classical complement cascade on the nerve surface is therefore a logical treatment approach that may reduce acute morbidity, disability, and mortality and increase the chances of better long‐term outcomes in patients with GBS.

ANX005 is a fully humanized, full‐length recombinant immunoglobulin G4 (IgG4) monoclonal antibody against C1q that selectively and fully inhibits C1q binding to surface‐bound antibodies and other substrates to prevent classical pathway activation [15, 19]. Immediate inhibition of and reduction in neuroinflammation and antibody‐mediated nerve damage during the disease's early phase, without affecting later‐stage natural recovery processes, may provide more optimal efficacy than current standard of care [13]. Importantly, selective inhibition of C1q and its specific role in amplifying antibody‐mediated disease leaves the alternative and lectin complement pathways intact to perform their normal clearance and host defense functions.

The current study with ANX005 is a Phase 1 trial in patients with GBS, designed to evaluate the safety, tolerability, pharmacokinetics (PK), and pharmacodynamics (PD) of ANX005. Clinical outcomes were also evaluated as exploratory endpoints, and their relationship with baseline characteristics was assessed in post hoc analyses.

## 2 Materials and Methods

### 2.1 Study Design and Intervention

ANX005‐GBS‐01 was a Phase 1, randomized, double‐blind, placebo‐controlled, single‐center dose‐escalation study of ANX005 in patients recently diagnosed with GBS.

The study was originally designed as an integrated two‐part trial, with the first phase evaluating the safety, tolerability, and PK of escalating doses of ANX005 in approximately 50 patients over 8 weeks and the second phase evaluating the efficacy of two dose regimens of ANX005 in approximately 126 patients over a 26‐week period. Only the first phase was conducted and completed, while the second phase was instead conducted as a stand‐alone Phase 3 study (NCT04701164). In the Phase 1 study, patients were enrolled in escalating dose cohorts (single or two infusions), each of which guided the dose and infusion approach in subsequent cohorts.

After obtaining an informed consent form (ICF), patients were screened and remained hospitalized until discharged into home care. ANX005 administration occurred within 48 h of screening and no later than 10 days after the onset of GBS‐related weakness. Patients were enrolled at one site, the National Institute of Neuroscience and Hospital (NINS) in Dhaka, Bangladesh, which is the national and public tertiary care hospital for GBS and the single largest contributor of the first 1000 patients enrolled in the International GBS Outcome Study (IGOS) [7]. Due to the high incidence of GBS in Bangladesh and the center's referral status, the NINS treats over 20 GBS patients per month. Therefore, this facility has unparalleled experience in anticipating and treating GBS‐related complications and provides daily in‐hospital full observation and multidisciplinary services for at least 4 weeks, the active phase of the disease. The site was recently awarded a center of excellence designation by the International GBS/Chronic Inflammatory Demyelinating Polyneuropathy (CIDP) foundation.

Patients were randomized to receive ANX005 or placebo in escalating doses as a single intravenous (IV) infusion on Day 1 or as two IV infusions of ANX005 75 mg/kg or placebo on Days 1 and 8 in seven sequential cohorts. Patients received the following doses of ANX005: 3, 9, 18, 36, 75, 75 (2 infusions), or 100 mg/kg, or matching placebo in addition to supportive standard‐of‐care medical treatment for GBS. Dose escalation did not exceed a three‐fold increase from the preceding cohort dose level, with a maximum dose of 100 mg/kg per infusion Patients were followed weekly (Weeks 1, 2, 3 and 4) after their first dose, with a final visit at Week 8.

Exposure to ANX005 3 and 9 mg/kg was limited to two or three patients, as these two dose levels were anticipated to provide less than 24 h of complement inhibition. All other cohorts enrolled at least eight patients, with at least six patients receiving ANX005 and at least two receiving placebo. ANX005 was administered at a slow initial infusion rate (0.33–0.75 mg/kg/h) to prevent infusion‐related reactions (IRRs), which were seen in a healthy volunteer study of ANX005 and deemed related to solution‐phase activation of C1q by ANX005 during the target saturation phase of infusion. IRRs involved transient complement‐mediated rash due to peripheral vasodilation and increased vascular permeability and were on occasion accompanied by transient changes in vitals akin to what is observed with the use of approved monoclonal antibodies [20]. After the first two cohorts, patients received premedication with an antihistamine and acetaminophen ≤ 2 h prior to the start of IV infusion, which was considered effective in the reduction of IRRs. The use of corticosteroids was not recommended because of their potential to negatively impact recovery from GBS, although corticosteroids as well as additional or alternative antihistamines or other IRR‐precaution medications were acceptable based on investigator discretion.

Long‐term complement inhibition is often achieved with meningococcal vaccination or antibiotic prophylaxis since it may predispose patients to infections with community acquired encapsulated bacteria, particularly, *Haemophilus influenzae* , *Streptococcus pneumoniae* , and *Neisseria meningitidis* [21]. However, due to the acute nature of the disease, the limited duration of complement inhibition (during which patients were hospitalized for observation), and the selective targeting of the classical pathway that leaves the lectin and alternative pathways intact, patients were not provided vaccination or antibiotic prophylaxis.

The single study site received institutional review board (IRB) approval from the International Center for Diarrheal Disease Research, Bangladesh (ICDDR,B), and this study was conducted in compliance with the Declaration of Helsinki and International Council for Harmonization Good Clinical Practice. An independent data and safety monitoring committee reviewed the safety of the patients in the study.

The primary objective of this study was to assess the safety, tolerability, PK, and PD of ANX005 as a first‐line monotherapy for GBS. A placebo‐controlled trial excluding standard of care with IVIg or PE was necessary to properly evaluate ANX005 in this first‐in‐human study. Bangladesh was chosen due to its high incidence of GBS, and the NINS was selected as an internationally recognized research site with extensive experience in providing care to GBS patients. Ethical considerations were aligned with the Declaration of Helsinki, as patients were enrolled only if they did not have access to IVIg or PE, ensuring that no one was denied treatments they would otherwise receive [22]. All patients received immediate supportive care. While IVIg and PE are the standard treatment for patients with GBS, many individuals continue to experience severe weakness and prolonged recovery periods [1, 10]. There have been no adequate placebo‐controlled trials of IVIg in patients with GBS, limiting IVIg's utility as a comparator in clinical studies [10]. Due to the potential interference of IVIg with the PK and PD of ANX005, monotherapy with ANX005 was deemed more informative for the study of ANX005 in patients with GBS.

### 2.2 Patient Population

Eligible patients included male and female individuals ≥ 18 years of age with a recent diagnosis of GBS according to the National Institute of Neurological Disorders and Stroke (NINDS) Diagnostic Criteria for GBS. Patients were also required to have an onset of GBS‐related weakness ≤ 10 days prior to infusion and a GBS‐Disability Score (GBS‐DS) of 3, 4, or 5 at screening and at Day 1 prior to infusion. Patients were excluded if they met any of the following criteria: previous treatment or intended future treatment with either PE or IVIg; diagnosis with a GBS variant, including Miller Fisher syndrome, Bickerstaff's encephalitis, and overlap syndromes; GBS‐related weakness that had improved since onset of symptoms or hospitalization; or history of prior GBS episodes.

### 2.3 Objectives and Endpoints

The primary objectives of this study were to evaluate the safety and tolerability of a single dose of IV ANX005 and establish the maximum tolerated dose (MTD) and the optimal biological dose of ANX005 in patients with GBS.

The primary safety endpoint was the incidence of treatment‐emergent adverse events (TEAEs) and serious adverse events (SAEs), which were assessed throughout the study according to the National Cancer Institute's Common Terminology Criteria for Adverse Events (NCI‐CTCAE) version 4.03. Dose‐limiting toxicities were defined as AEs of ≥ Grade 3 that occurred within 48 h of infusion and were determined by investigators to be related to treatment. The sponsor developed a grading system to assess IRRs based on the extent of hyperemia of the skin that can easily be observed: Grade 1 (rash < 20% body surface area [BSA]), Grade 2 (rash ≥ 20% and ≤ 50% BSA), Grade 3 (> 50% BSA ± signs and symptoms), or Grade 4 SAEs (including hypotension and tachycardia). Laboratory evaluations included hematology, serum chemistry (alanine aspartate aminotransferase [AST] and aminotransferase [ALT]), and urinalysis.

The secondary objectives were to assess the PK profile of ANX005 in patients with GBS and evaluate the efficacy of ANX005 using the GBS‐DS, MRC sum score, Overall Neuropathy Limitations Scale (ONLS), Fatigue Severity Scale (FSS), Rasch‐built Overall Disability Score (R‐ODS), and duration of mechanical ventilation utilization and intensive care unit (ICU) stay.

Exploratory PK and PD assessments included complement‐related and other biomarkers in serum (neurofilament light chain [NfL], cholesterol) and CSF (sphingomyelin [SM]), which were evaluated for their possible correlation with the PK profile of ANX005 and efficacy outcomes. Ex vivo PD measures of C1q functional activity were obtained with a standard 50% hemolytic complement assay (CH50), which determines the ability of C1q in patient serum samples to trigger classical complement‐mediated lysis of antibody‐sensitized red blood cells. This functional measure depends on the activity of all complement components in the classical pathway, including C1q, C4, C3, and C5‐C9; full CH50 inhibition thus demonstrates blockade of the entire classical pathway. sNfL was analyzed by patient neurotype status (AMAN vs. AIDP). Outcome measures were assessed at each visit and used to determine an optimal dose for ANX005.

Exploratory efficacy was evaluated in patients within the first five cohorts of the study, before the initial blind was broken, and was based on achievement of 1–3 weeks of C1q inhibition, representing the time of disease most likely to involve autoantibody‐driven complement activation. The efficacy evaluation, therefore, included eight placebo patients pooled from the first five cohorts of placebo (3–75 mg/kg) versus patients from the three active cohorts (18–75 mg/kg).

Free ANX005 serum concentrations were assessed throughout the study, namely, at the start of infusion, end of infusion, and 12 and 48 h after each infusion; on study Days 8, 15, 22, and 29; and at the end of study. Free ANX005 was also assessed in the CSF, collected at study screening and on either Day 5 or 8 after administration of ANX005. Free C1q, total CH50, and NfL sampling procedures varied with each cohort but were generally performed according to the ANX005 serum sampling schedule.

### 2.4 Statistical Analysis

Approximately 50 patients were enrolled in this study. This sample size was determined by clinical and practical considerations rather than statistical. Descriptive statistics were used to compare changes in biomarkers for patients receiving ANX005 and placebo. A proportional odds (PO) logistic regression model was also used to analyze clinical benefit in this study. This model has been used in an earlier GBS study [11] and may be of particular value in future trials. As opposed to a dichotomy, this analysis utilizes a range of outcomes that ANX005 might alter and more fulsomely captures the benefit of treatment to patients. For the purpose of this analysis, GBS‐DS was collapsed into three categories: Grades 0–1 (good state), 2–3 (disabled), and 4, 5, or 6 (severely disabled or death). The analysis included patients advancing all the way to a good state of health as well as any patient improving from severe disability to better health, providing a single statistic—the common odds ratio. By this measure, the PO represents overall improvement to a better state of health and provides greater statistical power beyond a dichotomized approach. Since no placebo‐treated patients advanced to a good state of health in this study and the odds ratio cannot use zero as a denominator, for the purpose of calculation, one placebo patient was imputed into this category (thereby disadvantaging the study drug).

Mann–Whitney testing was used to compare change from baseline in ONLS for patients receiving ANX005 and placebo. Least‐squares (LS) mean values and Spearman's rank correlation coefficient were used to evaluate the change in MRC sum score, and a cluster analysis was performed to compare muscle strength improvement over time.

## 3 Results

### 3.1 Patient Population

**FIGURE 1:**
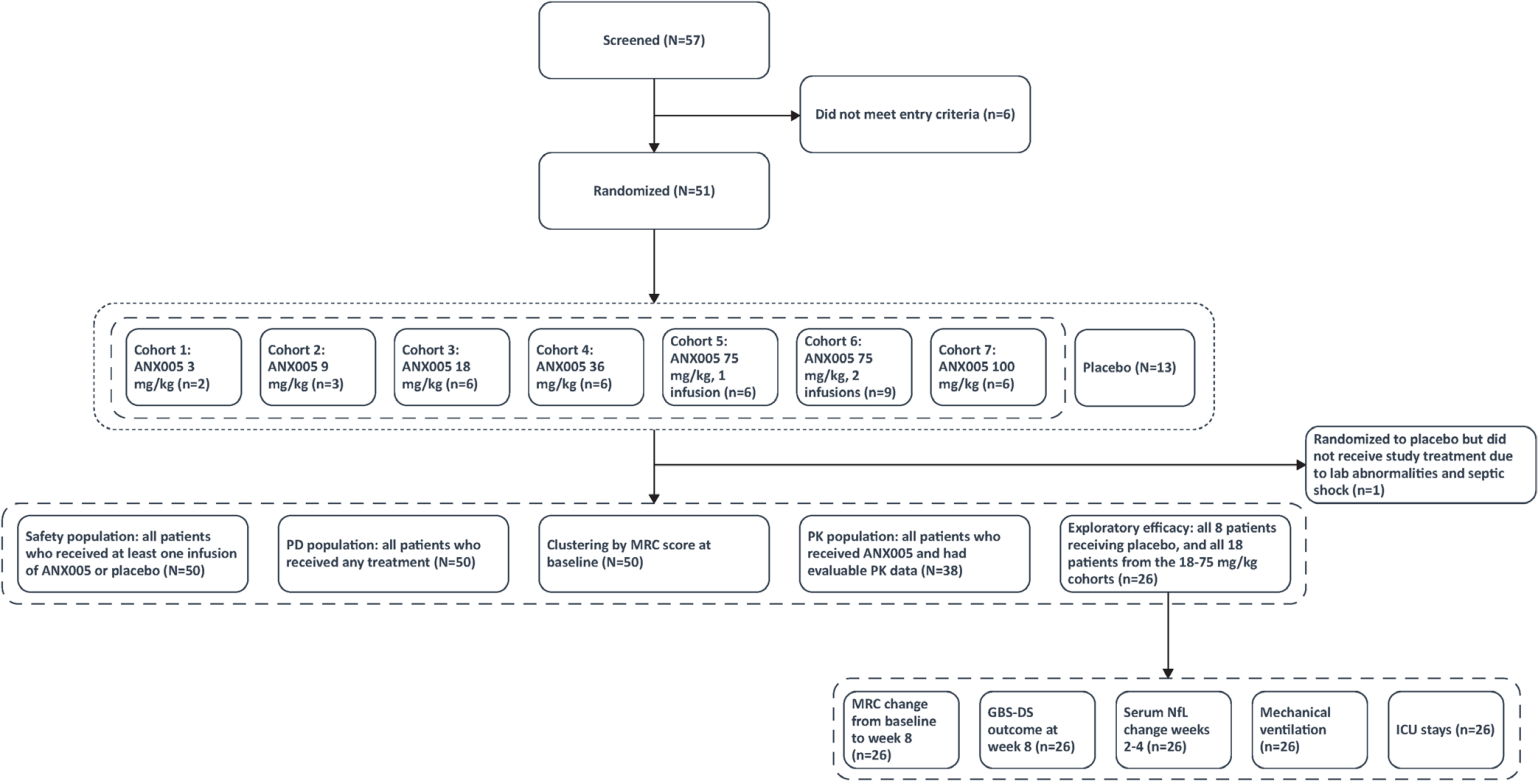
Patient populations and analyses. GBS‐DS, Guillain–Barré Syndrome‐Disability Score; ICU, intensive care unit; MRC, Medical Research Council; NfL, neurofilament light chain; PD, pharmacodynamics; PK, pharmacokinetics.

Between April 2018 and September 2019, a total of 57 patients were screened, of whom 51 were randomized to receive either ANX005 (*n* = 38) or placebo (*n* = 13) in one of seven cohorts (Figure 1). Fifty of the 51 randomized patients received study treatment (38 ANX005, 12 placebo); one patient was randomized to placebo but did not receive study treatment. All 50 patients who received study treatment completed the study.

The safety analysis population included all patients who received at least one infusion of ANX005 or placebo (*N* = 50). PK analysis involved all patients who received ANX005 and had evaluable PK data (*N* = 38), and PD analysis included all patients who received any treatment (*N* = 50). Exploratory efficacy analysis included patients in the first five cohorts, during which the initial blind was maintained, including all eight patients receiving placebo and all 18 patients from the 18–75 mg/kg cohorts, which provided 1–3 weeks of complement inhibition, covering the progressive phase and nadir of GBS.

The mean age of study patients was 31 years (range, 18–61 years; Table 1). All patients were of Asian race, most were relatively young with an unremarkable medical history, and the majority were male (64%, 32/50). Overall, the mean body mass index (BMI) was 22.5 kg/m^2^ (range, 15.3–31.8 kg/m^2^). There were no noteworthy differences in demographic characteristics among cohorts.

**TABLE 1:** Baseline characteristics and demographics (*N* = 50).

Parameter	ANX005 (*n* = 38)	Placebo (*n* = 12)	Overall (*N* = 50)
Age, years, mean (range)	31 (18–61)	33 (18–52)	31 (18–61)
Gender, *n* (%)			
Male	23 (61)	9 (75)	32 (64)
Female	15 (39)	3 (25)	18 (36)
BMI, kg/m^2^, mean (range)^a^	22.6 (15.3–31.8)	22.4 (16.9–30.0)	22.5 (15.3–31.8)
GBS‐DS, *n* (%)			
GBS‐DS 3	4 (11)	1 (8)	5 (10)
GBS‐DS 4	30 (79)	10 (83)	40 (80)
GBS‐DS 5	4 (11)	1 (8)	5 (10)
MRC sum score, mean (range)	15.3 (0–44)	16.3 (0–28)	15.5 (0–44)
NfL, pg/mL, mean	970.9	803.9	930.8
Time from symptom onset to randomization, days, mean (range)	8.1 (5–10)	7.1 (4–10)	7.8 (4–10)

Abbreviations: BMI, body mass index; GBS‐DS, Guillain–Barré Syndrome‐Disability Score; MRC, Medical Research Council; NfL, neurofilament light chain.

^a^ For BMI measurements, *n* = 37 for ANX005 and *n* = 11 for placebo.

As measured by baseline muscle strength and GBS‐DS, all patients had weakness in both upper and lower limbs, and most patients had pure motor weakness (no sensory deficit). In addition, almost all randomized patients had high levels of serum NfL (sNfL) as described in Table 1. Known baseline prognostic factors of GBS such as muscle strength and time until onset of weakness were reasonably evenly distributed between treatment and placebo groups. The mean ± SD time from onset of motor/muscle weakness to randomization was 8.1 ± 1.43 days (8.0 days) in the ANX005 cohorts and 7.1 ± 2.31 days (7.0 days) in the placebo cohorts. The mean ± SD GBS‐DS score at baseline was 4.0 ± 0.46 in the ANX005 cohorts and 4.0 ± 0.43 in the placebo cohorts. Overall, MRC sum scores at baseline were similar between the ANX005 and placebo groups (15.3 vs. 16.3, respectively). Baseline median sNfL was elevated in all patients, with higher levels (742.0 pg/mL) observed in those classified as AMAN (*n* = 30; 75%) versus AIDP (108.6 pg/mL, *n* = 10; 25%), and was distributed evenly across treatment arms. The Erasmus GBS Respiratory Insufficiency Score (EGRIS), which is used to predict the risk of mechanical ventilation, was 4.2 ± 0.87 in the ANX005 cohorts and 4.4 ± 0.79 in the placebo cohorts, suggesting that patients were at high risk of being ventilated. The modified Erasmus GBS Outcome Score (mEGOS), which is used to characterize prognosis, could not be calculated without diarrhea within 4 weeks preceding muscle weakness onset. Baseline characteristics and demographics were similarly distributed among the combined ANX005 dosing cohorts of 18, 36, and 75 mg/kg and placebo (Table 2).

**TABLE 2:** Baseline characteristics and demographics in the combined ANX005 dosing cohorts of 18, 36, and 75 mg/kg and placebo (*n* = 26).

Parameter	ANX005 (*n* = 18)	Placebo (*n* = 8)
Age, years, mean (range)	32 (18–61)	30 (19–43)
Gender, *n* (%)		
Male	11 (61)	6 (75)
Female	7 (39)	2 (25)
BMI, kg/m^2^, mean (range)^a^	21.2 (15.3–30.3)	21.8 (16.9–30.0)
GBS‐DS, *n* (%)		
GBS‐DS 3	1 (6)	0 (0)
GBS‐DS 4	14 (77)	6 (75)
GBS‐DS 5	3 (17)	2 (25)
MRC sum score, mean (range)	15.1 (0–40)	14.4 (0–24)
NfL, pg/mL, mean	1039	1165
Time from symptom onset to randomization, days, mean (range)	4.7 (1–9)	4.5 (1–7)

Abbreviations: BMI, body mass index; GBS‐DS, Guillain–Barré Syndrome‐Disability Score; MRC, Medical Research Council; NfL, neurofilament light chain.

^a^ For BMI measurements, *n* = 17 for ANX005 and *n* = 7 for placebo.

### 3.2 Safety

No patients died during the study, and no dose‐limiting toxicities were observed in any cohort (up to 100 mg/kg). As such, the MTD was not reached under this protocol.

All patients in the safety analysis population (*N* = 50) experienced at least one TEAE, which was determined to be related to treatment in 76% (29/38) of ANX005 patients and 17% (2/12) of placebo patients (Table 3). The incidence of ≥ Grade 3 TEAEs was similar between patients receiving ANX005 and placebo (21% and 25%, respectively), and no ≥ Grade 3 TEAEs were determined to be related to treatment. IRRs were the most commonly reported related TEAEs, seen in 76% (29/38) of patients receiving ANX005 and 8% (1/12) of patients on placebo. All IRRs were classified as a Grade 1 (66% [19/29]) or Grade 2 (34% [10/29]) skin rash that spontaneously and completely resolved without sequalae over time. In most patients who experienced a rash, the event occurred during infusion of the first 15 mg/kg of ANX005 during the target saturation phase. Additional occurrences of rash could appear in this same period of treatment or occasionally at later times during the infusion. The incidence of SAEs was lower in patients receiving ANX005 than in patients receiving placebo (5% and 25%, respectively), and there were no SAEs considered related to treatment. No infections with encapsulated organisms were reported. One patient (100 mg/kg ANX005) discontinued treatment due to a TEAE (back pain) that was not considered related to study treatment. Four patients in the 75 mg/kg × 2 group did not receive the second infusion of ANX005, as their clinical condition potentially prevented the detection or interpretation of a safety signal during the second infusion.

**TABLE 3:** Overall safety summary (*N* = 50).

Parameter, *n* (%)	ANX005 (*n* = 38)	Placebo (*n* = 12)
Any TEAE	38 (100)	12 (100)
Related TEAE	29 (76)	2 (17)
Infusion‐related TEAE	29 (76)	1 (8)
≥ Grade 3 TEAE	8 (21)	3 (25)
Related ≥ Grade 3 TEAE	0 (0)	0 (0)
TEAE leading to ANX005 discontinuation	1 (3)	0 (0)

Abbreviation: TEAE, treatment‐emergent adverse event.

Serum ALT levels followed the monophasic course of GBS in both treatment groups. Overall, 48% of patients had elevated ALT at baseline (mean ± SD, 38.45 ± 28.52 IU/L), which increased to 76% at Day 8 (mean ± SD, 124.51 ± 278.07 IU/L) and returned to baseline levels at Day 56 (mean ± SD, 58.63 ± 31.67 IU/L). Peak ALT levels were similar between patients receiving placebo and ANX005 (median ALT, 2.8× ULN vs. 2.5× ULN, respectively; *t* test, *p* = 0.63). Elevated serum ALT levels correlated with an AST/ALT ratio < 1.0 at all timepoints and returned to baseline at Day 56.

### 3.3 PK and PD

**FIGURE 2:**
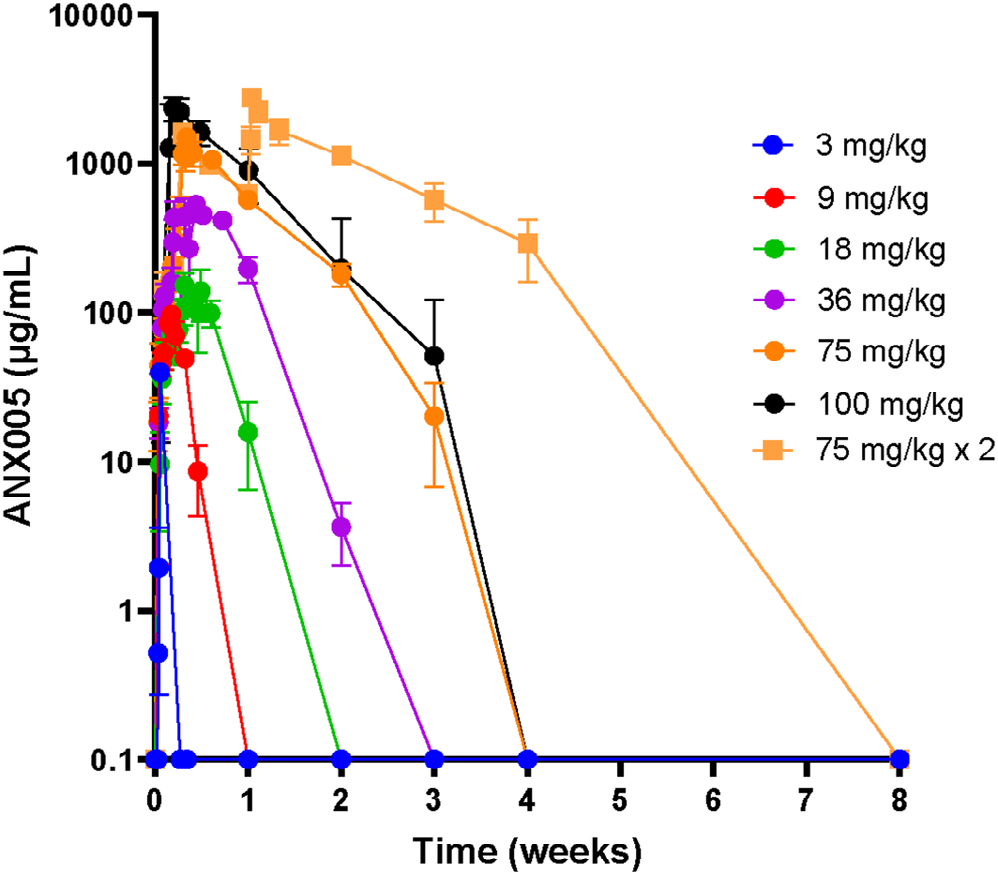
Mean free ANX005 in serum following single doses (*n* = 38).

**FIGURE 3:**
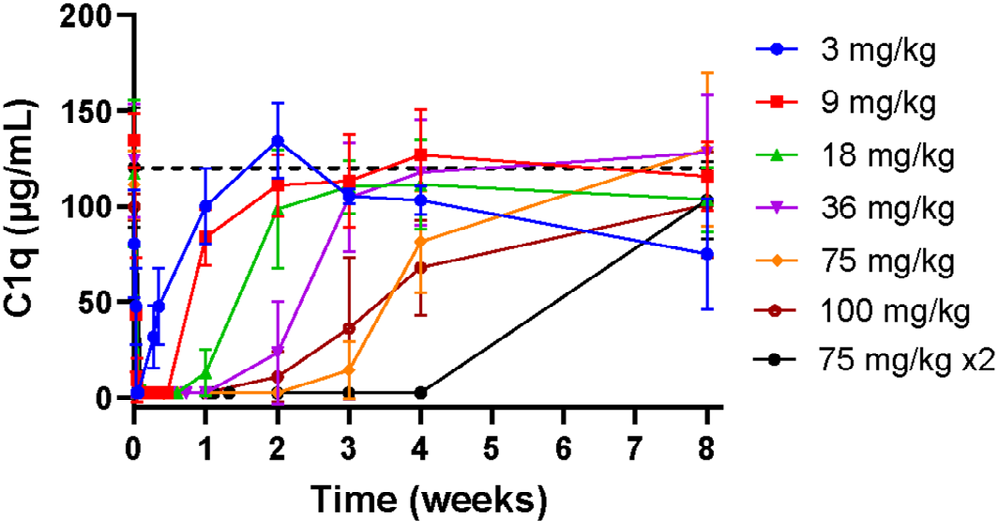
Free C1q levels in serum (*n* = 38).

**FIGURE 4:**
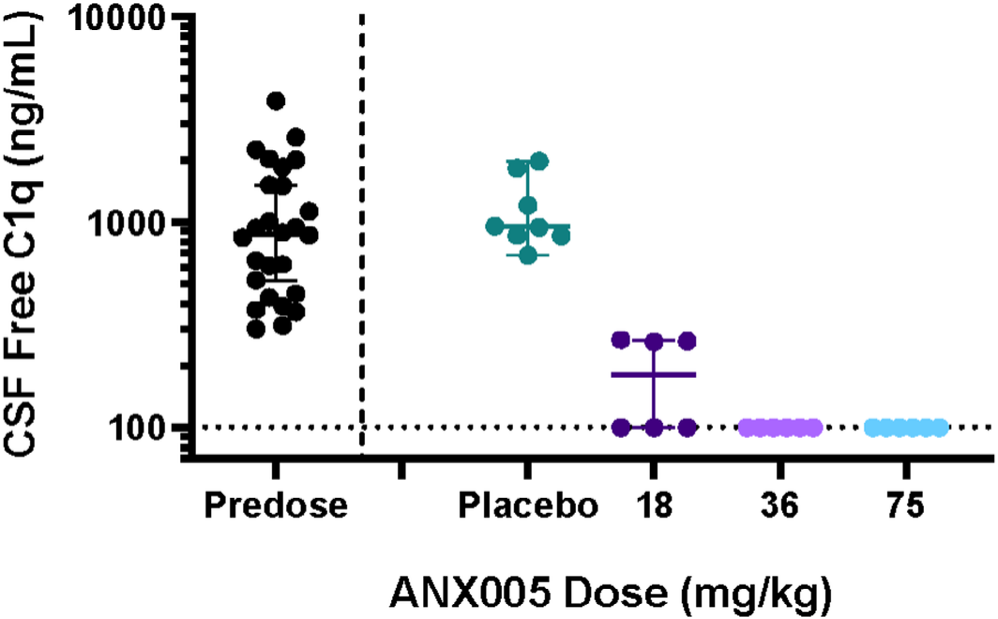
Free C1q levels in CSF by treatment (*n* = 26). CSF, cerebrospinal fluid.

Across dosing cohorts of 3–100 mg/kg in the PK set (*N* = 38), ANX005 exposure increased in a nonlinear and greater than dose‐proportional manner, as expected for a monoclonal antibody undergoing target‐mediated drug disposition (Figure 2). ANX005 exposure did not appear to be influenced by age, sex, body weight, or BSA. Functional activity of C1q was measured by standard CH50 analysis and was found to mirror ANX005 drug levels in all patients, with full inhibition whenever free drug was measurable (data not shown). Similarly, free C1q serum concentrations demonstrated an inverse correlation with free ANX005 serum concentrations in all dose cohorts (Figures 3 vs. 2). Duration of C1q inhibition increased with increasing ANX005 dose and serum exposure. Patients receiving a single dose of 18 or 36 mg/kg demonstrated complement inhibition for 1–2 weeks. The 75 and 100 mg/kg cohorts exhibited complete C1q inhibition for more than 2 weeks, and complete C1q inhibition was observed through 4 weeks in patients receiving two doses of 75 mg/kg. In the CSF, partial to full occupancy of C1q was observed in patients receiving 18 mg/kg, and full occupancy was observed in all patients receiving ≥ 36 mg/kg, indicating full C1q suppression (Figure 4) Inhibition of C1q in the CSF is thought to be important due to disease involvement of peripheral nerve roots and spinal nerves that are immersed in CSF as they exit the spinal cord, substantially contributing to the symmetrical weakness observed in GBS patients.

**FIGURE 5:**
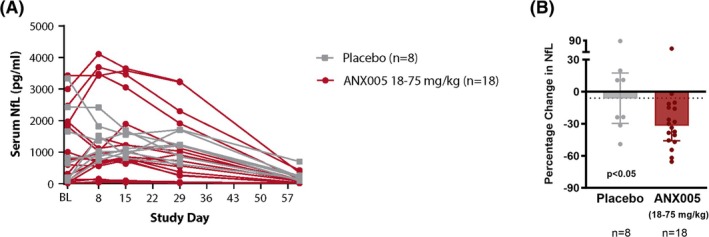
(A) NfL serum levels Weeks 2–4 in placebo‐ versus ANX005 18–75 mg/kg‐treated patients (*n* = 26). (B) Percentage change in NfL levels Weeks 2–4 in placebo‐ versus ANX005 18–75 mg/kg‐treated patients (*n* = 26). NfL, neurofilament light chain.

Baseline NfL levels covered a broad range, dependent on the timing of the disease course relative to hospital entry and neurotype of the disease. On average, NfL peaked by Day 15 in nearly all patients, consistent with the monophasic nature of GBS. Compared to placebo, treatment with ANX005 doses of 18–75 mg/kg led to a significant reduction in NfL levels from peak (Week 2) to Week 4 (*p* < 0.05, Figure 5A,B). At Week 8, NfL levels converged toward the normal range in both ANX005 and placebo groups. CSF levels of SM (csfSM), a diagnostic marker of demyelinating disease, and cholesterol, the predominant lipid of myelin, were highly correlated (*r* = 0.95, *p* < 0.0001). Both levels were elevated at baseline and continued to increase in patients who were functionally declining while decreasing in patients who were improving. sNfL and csfSM were elevated in all patients, reflecting the simultaneous presence of axonal and myelin damage in individual patients, while the sNfL/csfSM ratio was 3× higher in patients with AMAN versus AIDP (*p* = 0.01). High‐density lipoprotein (HDL) levels were initially suppressed in all patients, suggesting that the release of lipids during nerve damage saturated the carrier function of HDL. There was no signal for SM in serum.

### 3.4 Efficacy

Efficacy was explored in the first five cohorts during which the initial study blind was maintained. Exploratory efficacy included all placebo‐treated patients in these cohorts (*n* = 8) and all patients in the 18–75 mg/kg cohorts (*n* = 18), where C1q occupancy in the CSF was measurable, C1q inhibition was maintained between 1 and 3 weeks in the circulation, and tissue homeostasis returned to normal.

**FIGURE 6:**
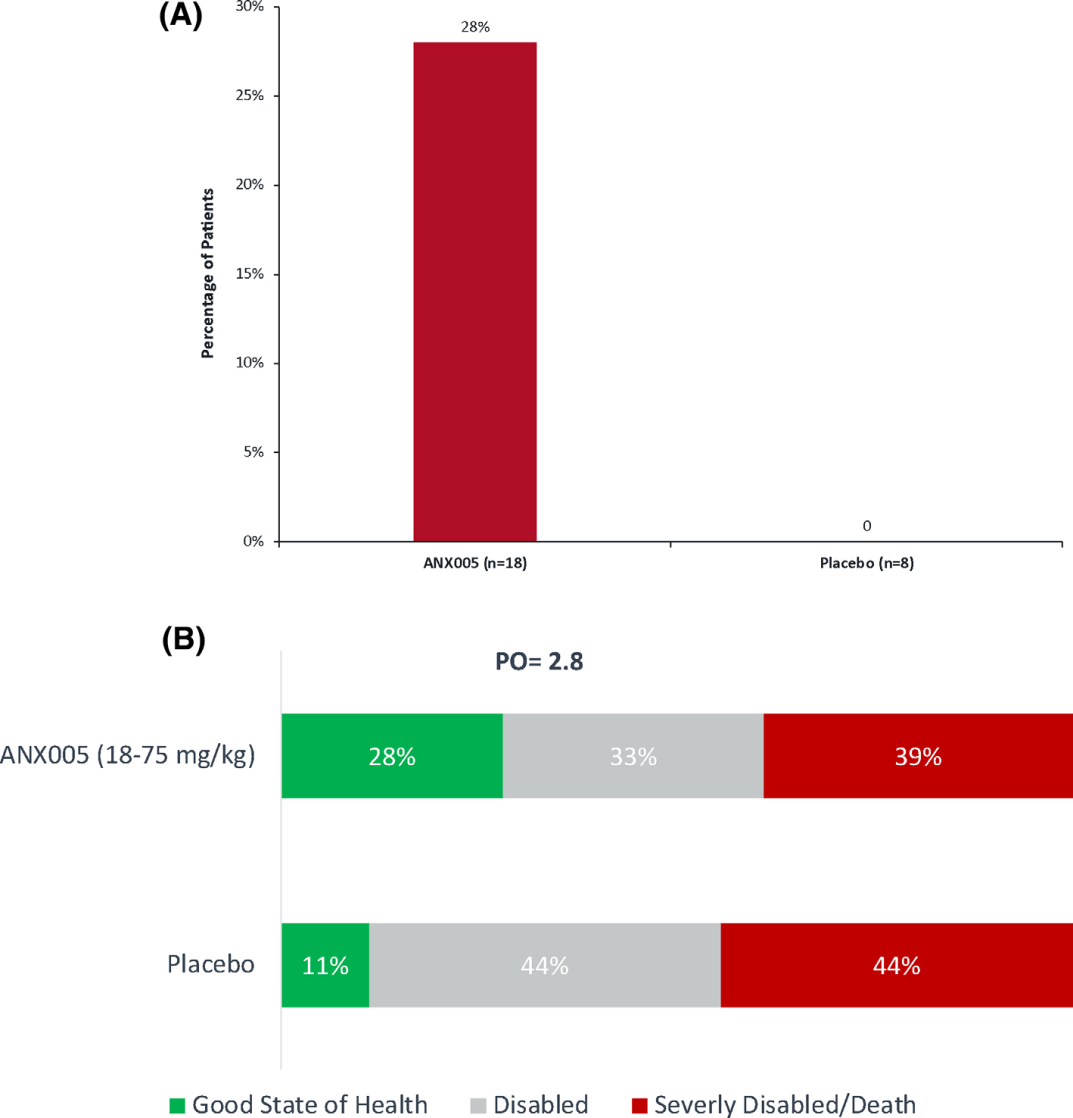
(A) Dichotomous efficacy outcome measures at 8 weeks: Ability to run (GBS‐DS 0, 1) or gain in GBS‐DS by ≥ 3 points (*n* = 26). (B) Proportional odds of improvement in GBS (*n* = 26). (0.53, 15.1), nonsignificant *p*‐value. To calculate PO, one patient is needed within each category. Given no placebo patients made it to the good state of health category, a conservative imputation method was used, and one placebo patient was added to the good state of health category to generate a PO. *N* = 27: ANX005 18–75 mg/kg (*n* = 18), placebo (*n* = 9). PO, proportional odds.

Among the exploratory efficacy group, 28% of ANX005‐treated patients (5/18) versus 0% of placebo patients (0/8) were able to run at the end of the study, involving an advancement of ≥ 3 points in GBS‐DS from baseline (Figure 6A). Notably, three patients who exhibited an improvement of three points or more had received a dose of 18 mg/kg, which provides 1 week of complement inhibition. Additionally, two patients had received a dose of 75 mg/kg, offering 2–3 weeks of complement inhibition. While the group size is too low for statistical significance, these differences are consistent with a clinically meaningful drug effect and represent two ways to analyze benefit on a dichotomous scale (percentage of patients regaining the ability to run or percentage of patients advancing ≥ 3 points on the GBS‐DS vs. those who did not).

The PO logistic regression model adjusted for baseline MRC sum score, age, and time from onset of weakness provided an odds ratio of 2.8 (*p* = 0.227), which indicates that the odds of improvement to a good state of health were 2.8 times higher for patients treated with ANX005 than placebo‐treated patients (Figure 6B).

The effect on improvement in GBS‐DS was also seen in an improvement in the ONLS score from baseline. At 8 weeks, ONLS scores in the ANX005 18–75 mg/kg group decreased from a median of 12 (95% CI, 10–12) at baseline to 6 (95% CI, 3–9). In the eight placebo patients, a decline in median ONLS at baseline from 10.5 (95% CI, 9–12) to 9 (95% CI, 2–12) was observed (Mann–Whitney, *p* = 0.11). There were no marked differences between ANX005 and placebo in FSS or R‐ODS and no dose‐related trends in these measures among the ANX005 dose groups.

The proportion of patients requiring ventilation was comparable between ANX005 (22% [4/18]) and placebo (25% [2/8]). Overall, the median (IQR) duration of ventilation was similar for ventilated patients receiving ANX005 (9.5 days [6.5–18.5]) or placebo (16.5 days [13.0–20.0]), which was observed in the absence of mortality. Incidence of ICU stays was comparable for patients receiving ANX005 (22.2% [4/18]) and placebo (25.0% [2/8]), and the median (IQR) duration of these stays was 20.5 (11.3–32.8) days in patients receiving ANX005 and 25.0 (22.0–28.0) days in those on placebo.

**FIGURE 7:**
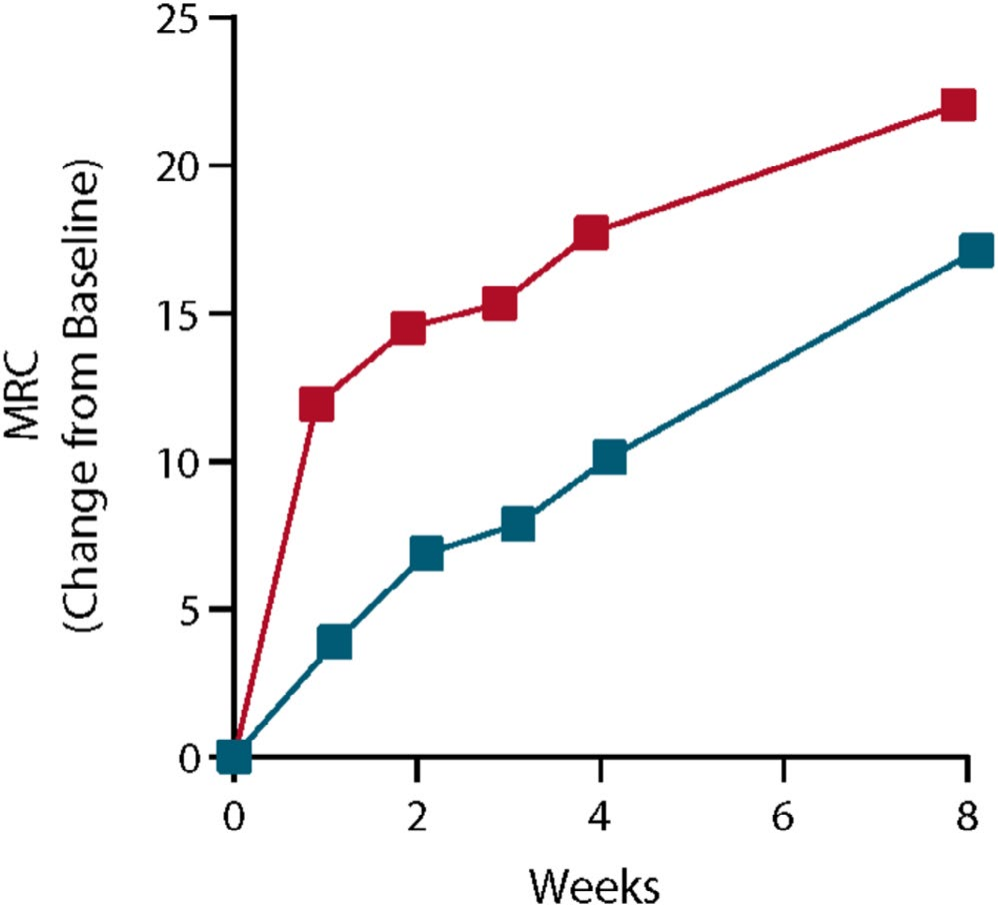
Change in MRC from baseline for ANX005 and placebo (*N* = 26). MRC, Medical Research Council.

There was a rapid, dose‐dependent increase in muscle strength as measured by MRC sum score within the first week of treatment, with larger improvements occurring in patients receiving ANX005 than in those receiving placebo. The mean change from baseline to Day 8 in MRC sum score was 11.8 for ANX005 and 3.9 for placebo (*p* = 0.09, Figure 7). The effect on the MRC sum score was also analyzed using the LS mean at Week 8, which showed an LS mean adjusted for baseline characteristics of 5.5 (*p* = 0.25).

**FIGURE 8:**
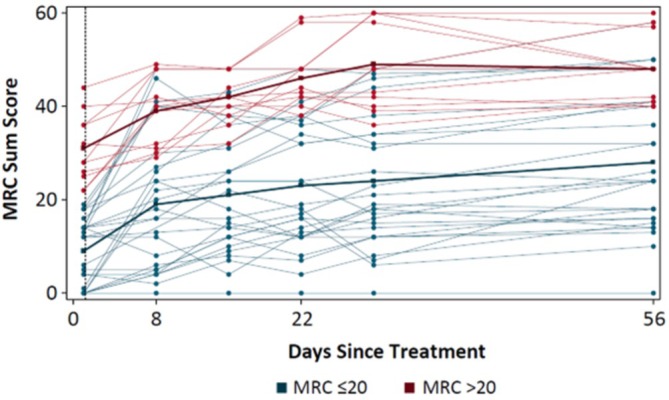
MRC sum score stratified by MRC score ≤ 20 and > 20 (*N* = 50). MRC, Medical Research Council.

Muscle strength and early improvement in MRC sum score from baseline are known to correlate with future functional recovery. Similarly, in this study, the change in MRC sum score from baseline to Week 1 correlated with the change in GBS‐DS at end of study (Spearman *r* = −0.518; *p* < 0.01; *n* = 25). Since improvement in muscle strength preceded an improvement in GBS‐DS, a cluster analysis using *k*‐means was performed to better understand the pattern of muscle strength improvement over time. Two clusters were identified by MRC sum score of ≤ 20 and > 20 at baseline. Analyzing patients by baseline MRC sum score ≤ 20 (lower cluster) and > 20 (upper cluster) in GBS‐01 revealed a correlation with function. A total of 14/16 (88%) patients with baseline MRC > 20 (range, 21–44) were able to walk independently or better (GBS‐DS ≤ 2) by Week 8 compared to only 7/34 (21%) with baseline MRC ≤ 20 (range 0–20) (*p* < 0.0001, Figure 8). Baseline NfL differed by cluster: it was 308 pg/mL (range, 13–1659 pg/mL) in patients with less affected muscle strength (upper cluster) versus 1224 pg/mL (range, 10–3946) in the lower cluster. These data highlight the importance of axonal damage to disease severity and of two prognostic factors to future functional recovery.

## 4 Discussion

This was the first randomized, placebo‐controlled, double‐blind trial investigating the utility of upstream classical complement inhibition in GBS. ANX005 was developed to selectively and immediately inhibit classical complement activation in blood, CSF, and tissue [15]. The approach in this study was designed to lessen disease severity and allow faster and more complete recovery.

ANX005 was examined with single infusion doses from 3 to 100 mg/kg ANX005 and two infusions of 75 mg/kg ANX005 1 week apart. An optimal administration schedule with an initial slow infusion rate of 0.33–0.75 mg/kg/h was empirically established during the conduct of this study, which allowed all patients to complete a single administration of ANX005 18–75 mg/kg. Adjustments to the administration were not deemed to impact the evaluation of efficacy based on PK and PD.

Overall, ANX005 administration was well‐tolerated in all dosing cohorts. There were no consistent patterns of abnormalities in safety parameters that appeared attributable to the study drug. There were no deaths, no treatment‐related SAEs, and no dose‐related AEs of concern. The MTD was not reached, as no dose‐limiting toxicities were observed at any dose level tested.

The dosing regimen included possible premedication with acetaminophen, corticosteroids, and antihistamines, with which 76% of patients receiving ANX005 exhibited one or more IRRs, typically during the initial phases of infusion. The incidence of IRRs appears related to initial target saturation, beyond which it was not dependent on dose, and all IRRs were transient and Grades 1–2. Transient increases in liver function tests (LFTs) were observed in both the ANX005 and placebo treatment groups and determined to be unrelated to treatment. These findings are consistent with previous studies, indicating that LFT abnormalities may be related to the GBS disease process [23, 24, 25, 26]. Overall, clinical lab abnormalities were frequent and pronounced across placebo and treatment groups, likely related to the severity of GBS seen in this study.

Across the dose range investigated, ANX005 exposure increased in a nonlinear and greater than dose‐proportional manner, as expected for a monoclonal antibody undergoing target‐mediated drug disposition [27]. The duration of C1q inhibition in blood and CSF increased with increasing ANX005 dose and exposure. Single doses in the range of 18 to 75 mg/kg defined the initial blinded stage of the study for exploratory efficacy analysis, as they provide between 1 and 3 weeks of inhibition, allowing C1q function to return to normal after the end of the active disease process.

In this analysis, surrogate objective biomarkers such as NfL and muscle strength as well as function using GBS‐DS and ONLS were assessed as exploratory endpoints. Compared to placebo‐treated patients, patients receiving single infusions of 18–75 mg/kg ANX005 had a significant reduction in sNfL between Weeks 2 and 4 (*p* < 0.05). Week 2 was chosen, as this was the approximate time of peak NfL. Elevated NfL levels in the serum or CSF of GBS patients indicate nerve damage, with higher levels being associated with more severe disease and a poorer prognosis, including longer hospital stays, slower recovery, and a greater likelihood of residual disability [28, 29, 30]. The early reduction in NfL seen with ANX005 correlated with the ability of patients to improve in GBS‐DS by Week 8. Further research to evaluate the use of NfL as a response biomarker is planned. There was a positive effect on GBS‐DS, which is used as the primary endpoint in GBS, and on ONLS, which measures motor function and impairment in performing daily tasks. Better scores were seen with ANX005 over placebo at Week 8 for both measures.

Additionally, patients receiving a single dose of 18–75 mg/kg ANX005 experienced rapid improvement in MRC sum score versus placebo, which correlated with better function at Week 8. Two clusters of improvement were observed, defined by their baseline MRC sum score of ≤ 20 and > 20. The highest NfL levels were seen in patients with poor muscle strength (MRC sum score ≤ 20) at baseline. While the 8‐week study period was relatively short, improvement in MRC sum score at Week 1 is an important prognostic indicator [5], suggesting that longer‐term benefits of ANX005 treatment are likely to be observed. Post hoc analyses of these exploratory endpoints suggest that inhibiting complement with ANX005 during the acute progressive phase of disease may be of benefit. The optimal dose of ANX005 and definitive evidence of its safety and efficacy will have to be established in future studies.

This study was conducted in Asian patients at an internationally recognized site in Bangladesh, a country in which GBS patients present with severe disease [7]. These results obtained in patients with a poor prognosis suggest that a beneficial effect of ANX005 in a broader or less severe GBS population is likely to be observed. Consistent with this suggestion, baseline prognostic factors such as muscle strength and NfL were found to be predictive of outcome in this study, as has been observed for patients outside Bangladesh [7].

Complement inhibition (anti‐C5) has been previously evaluated in GBS patients with little to no efficacy, and an IgG‐targeted therapy is also under evaluation (imlifidase) [31]. Key differences between inhibition of C1q versus C5 include (1) the ability of anti‐C1q to block classical complement activation at the start of the cascade, preventing cell surface deposition of C1q as well as surface deposition of downstream C4, C3, and C5–C9 activation products [15]. Inhibition of C5 still allows C1q and activated C4 and C3 products to accumulate on the cell surface, facilitating ongoing attack by macrophages through their complement‐recognition receptors, as well as C5 breakthrough activity due to the density of upstream complement surface accumulation [15, 32]; and (2) the ability of anti‐C1q to selectively inhibit the classical complement pathway, which is the main amplifier of antibody function and is key to the GBS disease process. While it fully blocks the classical cascade, anti‐C1q leaves the lectin and alternative pathways in place for their normal tissue clearance and repair function. Inhibition of C5, by contrast, inhibits the terminal cascade components for all three complement pathways.

While this study was not optimized to treat patients as early as possible, a thorough analysis of all available data showed an early and internally consistent impact of classical complement inhibition with ANX005 on markers of nerve damage, muscle strength at Week 1, and GBS‐DS and ONLS at Week 8. While some comparisons were statistically significant, results should be interpreted cautiously due to their exploratory nature, the small sample size, and the potential large impact of covariate adjustment.

Despite the use of well‐established immunotherapies, GBS continues to be a severe and potentially life‐threatening condition. The results of this study warrant additional investigation of a therapy that directly targets the underlying mechanism of nerve damage in GBS to meet a significant unmet medical need. The current study also provides data for stratification and analysis of the primary endpoint for an ongoing Phase 3 study of ANX005 in patients with GBS (NCT04701164).

## Conflicts of Interest

Quazi Deen Mohammad reports a consultancy/advisory role with Annexon Biosciences. Zhahirul Islam reports research funding from Fogarty International Center, National Institute of Neurological Disorders and Stroke of the National Institutes of Health, USA, and Annexon Biosciences. Nowshin Papri, Shoma Hayat, Israt Jahan, and Khan Abul Kalam Azad report no disclosures relevant to the manuscript. Dean R. Artis and Benjamin Hoehn are employees of Annexon Biosciences. Eric Humphriss is a former employee and shareholder of Annexon Biosciences. Ted Yednock is an employee of Annexon Biosciences and holds equity ownership in Annexon Biosciences. Henk‐André Kroon is an employee and shareholder of Annexon Biosciences.

## Data Availability

The data that support the findings of this study are available from the corresponding author upon reasonable request.
